# The efficacy of preoperative administration of gabapentin/pregabalin in improving pain after total hip arthroplasty: a meta-analysis

**DOI:** 10.1186/s12891-016-1231-4

**Published:** 2016-08-30

**Authors:** Yingdelong Mao, Lianguo Wu, Weiguo Ding

**Affiliations:** 1 Department of Orthopaedics, The Second Affiliated Hospital of Zhejiang Chinese Medical University, 318 Chaowang Road, Hangzhou, Zhejiang 310005 People’s Republic of China; 2 Department of Orthopaedics, Tongde Hospital of Zhejiang Province, 234 Gucui Road, Hangzhou, Zhejiang 310012 People’s Republic of China

**Keywords:** Gabapentin, Pregabalin, Total hip replacement, Meta-analysis

## Abstract

**Background:**

The purpose of this systematic review and meta-analysis of randomised controlled trials (RCTs) was to evaluate the pain control by gabapentin or pregabalin administration versus placebo after total hip arthroplasty (THA).

**Methods:**

In January 2016, a systematic computer-based search was conducted in the Medline, Embase, PubMed, CENTRAL (Cochrane Controlled Trials Register), Web of Science and Google databases. This systematic review and meta-analysis were performed according to the PRISMA statement criteria. The primary endpoint was the cumulative morphine consumption and visual analogue scale (VAS) scores at 24 and 48 h with rest or mobilisation. The complications of vomiting, nausea, dizziness and pruritus were also compiled to assess the safety of gabapentin and pregabalin. Stata 12.0 software was used for the meta-analysis. After testing for publication bias and heterogeneity across studies, the data were aggregated for random-effects modelling when necessary.

**Results:**

Seven studies involving 769 patients met the inclusion criteria. The meta-analysis revealed that treatment with gabapentin or pregabalin can decrease the cumulative morphine consumption at 24 h (mean difference (MD) = −7.82; 95 % CI −0.95 to −0.52; *P* < 0.001) and 48 h (MD = −6.90; 95 % CI −0.95 to −0.57; *P* = 0.118). Gabapentin or pregabalin produced no better outcome than placebo in terms of VAS score with rest at 24 h (SMD = 0.15; 95 % CI −0.17 to −0.48; *P* = 0.360) and with rest at 48 h (SMD = 0.22; 95 % CI −0.25 to 0.69; *P* = 0.363). There was no statistically significant difference between the groups with respect to the VAS score at 24 h postoperatively (SMD = 0.46; 95 % CI −0.19 to 1.11; *P* = 0.164) and at 48 h postoperatively (SMD = 1.15; 95 % CI −0.58 to 2.89; *P* = 0.193). Gabapentin decreased the occurrence of nausea (relative risk (RR), 0.49; 95 % CI 0.27–0.92, *P* = 0.025), but there was no significant difference in the incidence of vomiting, dizziness and pruritus.

**Conclusions:**

On the basis of the current meta-analysis, gabapentin or pregabalin can decrease the cumulative morphine consumption and decrease the occurrence of nausea; however, further trials are needed to assess the efficacy of pain control by gabapentin or pregabalin.

**Electronic supplementary material:**

The online version of this article (doi:10.1186/s12891-016-1231-4) contains supplementary material, which is available to authorized users.

## Background

Postoperative pain after total hip arthroplasty (THA) remains one of the most difficult types of pain to manage. Resuming ambulation as soon as possible after the operation can decrease the occurrence of deep venous thrombosis (DVT) and the economic cost of recovery [1, 2]. Many therapeutic modalities–ranging from nonsteroidal anti-inflammatory drugs (NSAIDs) to systemic opioids, acetaminophen, patient-controlled opioid analgesia and tramadol–have been used for postoperative pain management [3–7]. However, NSAIDs increase the occurrence of bleeding. Opioids increase the risk of nausea and vomiting as well as respiratory depression. Patient-controlled analgesia always provides inadequate analgesia for movement, which may delay hospital discharge. Acetaminophen alone provides pain relief, and thus, more opioids are required for rescue analgesia [8].

Contemporary postoperative pain management is aimed at enhancing pain relief and decreasing opioid consumption by combining analgesic drugs and techniques to reduce opioid-related complications. According to animal studies, pregabalin may reduce hyperalgesia related to inflammation or opioids [9, 10]. Gabapentin and pregabalin, referred to as gabapentinoids, are structural analogues of gamma-amino butyric acid, which is an anticonvulsant drug that possibly exerts its effects through voltage-dependent calcium channels. Gabapentin and pregabalin have similar antiallodynic and antihyperalgesic properties, which may be beneficial in controlling postoperative pain after THA. Many studies have compared gabapentin or pregabalin with placebos in managing pain after THA. However, there are no systematic reviews that evaluate the efficacy and safety of gabapentin or pregabalin for pain control after THA. We therefore searched electronic databases and conducted a systematic review and meta-analysis to identify the clinical outcome and safety of gabapentin or pregabalin in reducing pain after THA.

## Methods

### Search strategy

The following electronic databases were searched for relevant academic clinical trials comparing perioperative gabapentinoids (gabapentin and pregabalin) to a placebo for the management of pain after THA from inception to January 2016: Medline, Embase, PubMed, CENTRAL (Cochrane Controlled Trials Register), Web of Science and Google (Additional file 1). Both gabapentin and pregabalin have antiallodynic and antihyperalgesic properties, which may be beneficial in controlling postoperative pain after THA. Therefore, we incorporated these two drugs in this meta-analysis. The key words and medical subject heading (Mesh) terms included the following: gabapentin, pregabalin, pain control, total hip arthroplasty, total hip replacement, THA and THR. These key words and the corresponding MeSH terms were combined with the Boolean operators AND and OR. Furthermore, the reference lists of the identified literature were reviewed to identify any initially omitted studies, and no restriction was made on the language of the publication. Two reviewers independently searched the databases and filtered the relevant literature. Conflicts were resolved by the third reviewer. The full articles were screened to determine whether the articles fit the inclusion and exclusion criteria. Because this is a meta-analysis, no ethics committee or institutional review board approval was required.

### Inclusion criteria and study selection

The inclusion criteria were as follows: (1) randomised controlled trials (RCTs); (2) patients who underwent a primary THA; (3) interventions, including gabapentin or pregabalin, versus control (placebo or nothing); and (4) reported outcomes, including postoperative VAS pain with rest or mobilisation at 24 and 48 h, cumulative morphine consumption at 24 and 48 has well as the incidence of pruritus, vomiting, dizziness, and nausea. The article needed to include at least one of the outcomes mentioned above. We excluded studies of cadavers or artificial models. We also excluded non-RCTs, letters, comments, editorials, practice guidelines and other studies with insufficient data.

### Data abstraction and quality assessment

Duplicates were excluded using Endnote software, and two reviewers independently screened the titles and abstracts of the searched literature. Most of the articles were excluded on the basis of the topic of the article provided in the title or abstract, and disagreements about whether an article should be included were resolved by discussion or by a senior reviewer. Postoperative pain intensity was measured on a 100-point visual analogue scale (VAS). The 10-point VAS score and numerical rating scale were converted to a 100-point VAS score according to the reference. Data in other forms (i.e., median, interquartile range and mean ± 95 % CI) were converted to mean ± SD according to the Cochrane Handbook. If the data were not reported numerically, we extracted them using the “GetData Graph Digitizer” software from the published figures.

The following data were extracted and recorded in a spreadsheet: (1) the author’s name, demographic data about the number of patients in the gabapentin and control groups, the number of male patients in each group, the dose and time to administration of gabapentin and the anaesthesia method; (2) intraoperative and postoperative analgesia; and (3) the VAS score with rest or mobilisation at 24 and 48 h, the incidence of pruritus, vomiting, dizziness, sedation and nausea, and the cumulative morphine consumption at 24 and 48 h. Two reviewers independently scanned the quality of the eligible studies. Discrepancies were resolved by consensus after discussion, and a third reviewer participated in the debate to determine the final outcome if necessary. The risk of bias for each RCT was evaluated using the Cochrane Collaboration’s Risk of Bias Tool.

### Statistical analysis

Continuous outcomes such as the VAS score with rest or mobilisation at 24 and 48 h and the cumulative morphine consumption at 24 h and 48 h were expressed as the mean difference (*MD*) with the respective 95 % *CI*s. Discontinuous outcomes (i.e., the incidence of pruritus, vomiting, dizziness, sedation and nausea) were expressed as the relative risk (*RR*) with 95 % CIs. Statistical significance was set at *P* < 0.05 to summarise the findings across the trials. Risk of bias assessment for each involved article was conducted in light of the Cochrane Handbook for Systematic Reviews of Interventions using RevMan 5.30 software (The Cochrane Collaboration, Oxford, United Kingdom). The meta-analysis values were calculated by Stata, version 12.0 (Stata Corp., College Station, TX). Different gabapentin doses and dosing times in a single study were handled as subgroups within the study. Statistical heterogeneity was tested using the chi-squared test and I^2^ statistic. A chi-squared test scoring *I*^2^ > 50 % was considered suggestive of statistical heterogeneity. When there was no statistical evidence of heterogeneity, a fixed effects model was adopted; otherwise, a random effects model was chosen. Publication bias was tested by Begg’s test and was none if the *P* value obtained from Begg’s test is greater than 0.5.

## Results

### Search results

**Fig. 1:**
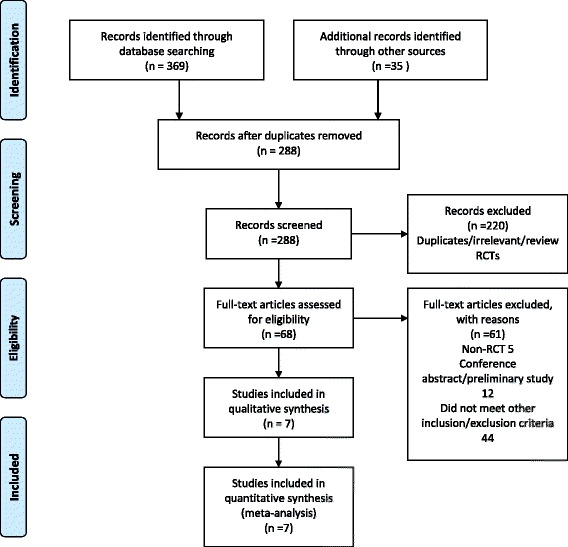
The flow diagram of the included studies

**Fig. 2:**
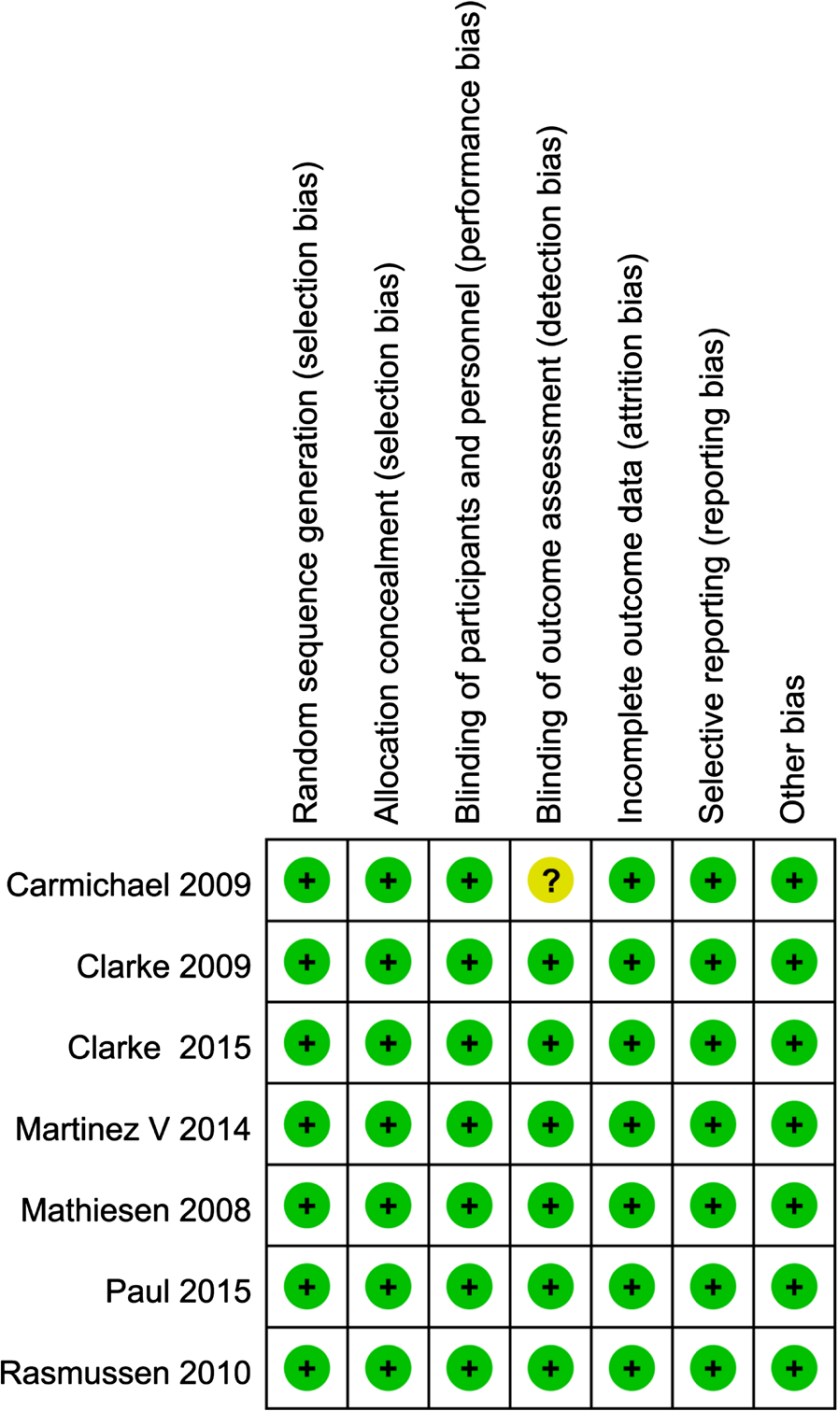
The detailed bias summary of each study

**Fig. 3:**
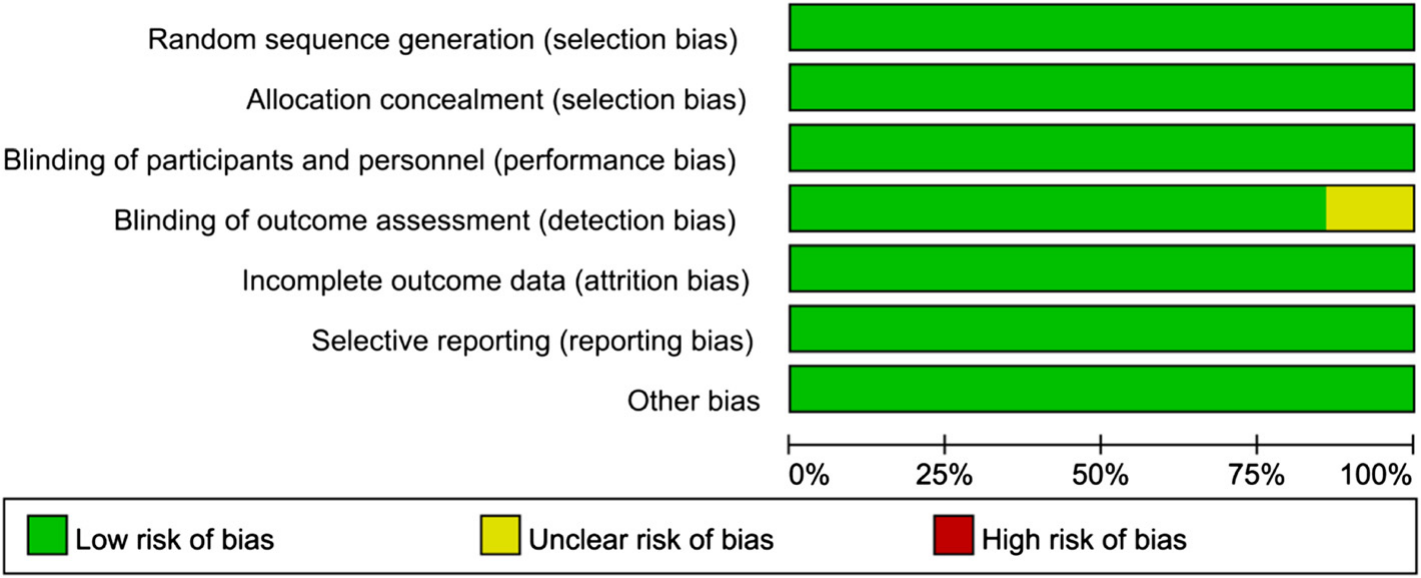
The bias summary concluded in the above graph

In the initial search, we identified 312 potentially relevant studies, of which 30 duplicates were removed by Endnote software (Fig. 1). According to the inclusion criteria, 276 studies were excluded after reading the titles and abstracts. Finally, we included seven clinical trials with 769 patients in the meta-analysis [11–17]. In the included studies, one trial used gabapentin in a different phase, and the groups were classified into preoperative administration and postoperative administration. Thus, the study was divided into two groups. The characteristics of the included studies are shown in Table 1. The number of patients ranged from 23 to 78. One study [14] performed THA using general anaesthesia, and the others performed THA by using spinal anaesthesia. In the included studies, a total of 621 THAs were performed, and the number of patients who received gabapentin, pregabalin or placebo was 150, 181 and 290, respectively. One article was published in 2008 [15]; one was published in 2009 [13] and the others were published from 2010 to 2015 [11, 12, 14, 16]. The participants in the five studies were mostly elderly, and the age of the patients ranged from 58.9 to 72 years. There were 266 male patients and 355 female patients. Four studies focused on the administration of pregabalin, and three studies on the administration of gabapentin for pain control after THA. The dose of pregabalin ranged from 150 to 300 mg/day preoperatively, and the dose of gabapentin ranged from 600 to 1200 mg/day preoperatively. The administration time for pregabalin and gabapentin was 1 and 2 h before surgery. Two studies performed the surgical procedure using the lateral approach for arthroplasty [15, 17], one study performed THA using posterior surgical approach [14] and the remaining studies did not state the approach used for performing THA [11–13, 16]. The postoperative analgesia included patient-controlled analgesia (PCA) and morphine or celecoxib. Details are shown in Table 2. All seven RCTs introduced randomisation, of which six trials were randomised by the computer-generated block method and one study was randomised by Randomization.com; only one trial did not imply blinding of outcome assessment. The risk of bias is shown in Figs. 2 and 3.

**Table 1:** The general character of the included studies

Clinical trial	Number of patients (G/C)	Mean age (G/C, yr)	Male/Female	anesthesia	Dose of gabapentin	Time to use the pregablin	Intraoperative analgesai	preoperative analgesia	Postoperative analgesics
Carmichael et al. [11]	23/24	59.1/61.3	22/25	spinal anesthetic	75 mg twice per day	2 weeks preoperative and for 3 weeks from the day of discharge	NS	400 mg celecoxib, 150 mg pregabalin and 1 g of acetaminophen	*
Clarke et al. [12]	83/79	60.2/60.1	82/80	spinal anesthesia	150 mg/day	2 h before surgery	Hypobaric bupivacaine 0.5 % (10 ml) with fentanyl 10 μg was injected	celecoxib	PCA
Martinez et al. [14]	35/38	64/64	45/28	general anesthesia	150 mg/day	before surgery	0.2 lg.kg 1 sufentanil followed by 2 mg.kg 1 propofol and 0.5 mg.kg 1	NS	PCA
Mathiesen et al. [15]	40/38	67/66	32/46	spinal anesthesia	300 mg/day	1 h before anesthesia	3 ml plain bupivacaine 5 mg ml	acetaminophen 1 g was given as premedication 1 h before anesthesia	PCA
Rasmussen et al. [17]	24/18	72/70	18/24	spinal anesthesia	1200 mg/day	1 h before anesthesia	3 ml plain bupivacaine (5mgml	NS	PCA
Paul et al. [16]	48/54	60.9/60.5	58/64	spinal anesthesia	600 mg/day	2 h before surgery	fentanyl 20 lg and 0.5 % or 0.75 % of bupivacaine	NS	PCA
Clarke et al. [13]	78/39	58.9/61.3 60.4/61.3	49/45	spinal anesthesia	600 mg/day	2 h before surgery	15 mg of 0.5 % hypobaric bupivacaine with 10 mg of fentanyl	acetaminophen 1000 mg per (p.o.), celecoxib 400 mg p.o. and dexamethasone 8 mg iv	PCA

*PCA* patient controlled anesthesia, *iv* intravenous, *p.o* postoperative, *NS* not stated

*pregabalin (75 mg twice per day), celecoxib (200 mg twice per day) and acetaminophen (1 g every 6 h) for 5 day

**Table 2:** The results of cumulative consumption and VAS score at 24 h and 48 h, NS, not applicable

Variables	Studies (n)	Patients (n)	*p*-value	Incidence		
				Mean difference (95 % CI) or risk ratio (95 % CI)	Heterogeneity *p*-value (I^2^)	Model
24 h cumulative consumption						
Overall	5	540	<0.001	−7.82 (−0.95, −0.52)	<0.001 (87.2 %)	random
Gabapentin	3	300	<0.001	−2.65 (−3.67, −1.63)	0.427 (0 %)	random
Pregabalin	2	240	<0.001	−19.42 (−11.72, −3.93)	<0.001 (59.9 %)	random
48 h cumulative consumption						
Overall	5	378	0.118	−6.90 (−0.95, 0.57)	<0.001 (93.7 %)	random
Gabapentin	3	258	1.000	0.00 (−7.69, 7.69)	<0.001 (94.5 %)	random
Pregabalin	2	120	<0.001	−33.02 (−45.86, −20.19)	0.989 (0.00 %)	random
VAS score with rest at 24 h						
Overall	5	451	0.360	0.15 (−0.17, −0.48)	0.017 (63.7 %)	random
Gabapentin	3	300	0.076	0.32 (−0.03, 0.67)	0.082 (55.3 %)	random
Pregabalin	2	151	0.486	−3.05 (−11.63, 5.53)	0.193 (41.1 %)	random
VAS score with rest at 48 h						
Overall	3	331	0.363	0.22 (−0.25, 0.69)	0.003 (78 %)	random
Gabapentin	2	258	0.049	0.41 (0.00, 0.81)	0.072 (62.1 %)	random
Pregabalin	1	73	0.106	NS	NS	NS
VAS score with mobilization at 24 h						
Overall	5	451	0.164	0.46 (−0.19, 1.11)	<0.001 (91 %)	random
Gabapentin	3	300	1.137	0.72 (0.23, 1.66)	<0.001 (93.1 %)	random
Pregabalin	2	151	0.803	−0.78 (−6.91, 5.35)	0.799 (0 %)	random
VAS score with mobilization at 48 h						
Overall	3	331	0.193	1.15 (−0.58, 2.89)	<0.001 (97.8 %)	random
Gabapentin	2	258	0.045	1.90 (0.04, 3.75)	<0.001 (97.2 %)	random
Pregabalin	1	73	<0.001	NS	NS	NS

### Results of the meta-analysis

#### Cumulative morphine consumption at 24 and 48 h

**Fig. 4:**
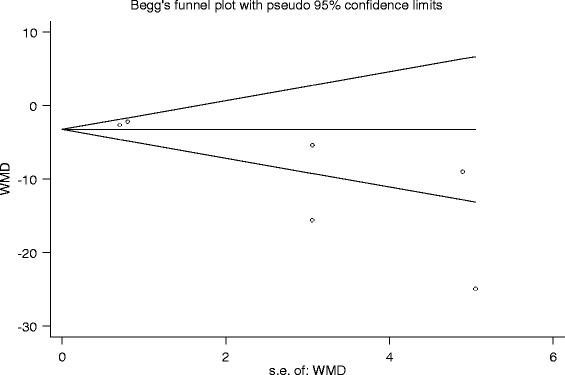
Begg’s funnel plot with pseudo 95 % CI

A total of five studies addressed the cumulative morphine consumption at 24 h and 48 h in the gabapentin or pregabalin and control groups. The results indicated that perioperative gabapentin or pregabalin can decrease the cumulative morphine consumption at 24 h (MD = −7.82; 95 % CI −0.95 to −0.52; *P* < 0.001) and 48 h (MD = −6.90; 95 % CI −0.95 to −0.57; *P* = 0.118, Table 2). Begg’s funnel plot is approximately asymmetrical and thus indicated that there is no publication bias between the included studies for the cumulative morphine consumption by 24 h (*P* = 0.133, Fig. 4).

Subgroup analyses were conducted to analyse the efficacy of gabapentin and pregabalin for pain control after THA. The results indicated that gabapentin can decrease the cumulative morphine consumption at 24 h (−2.65, (−3.67, −1.63), *P* < 0.001) and 48 h (0.00, (−7.69, −7.69), *P* < 0.001) with a significant difference (Table 2). Pregabalin can also decrease the cumulative morphine consumption at 24 h (−19.42 (−11.72, −3.93)) and 48 h (−33.02 (−45.86, −20.19)) with a significant difference (Table 2).

#### VAS score with rest

Only five studies with 451 patients provided a VAS score at 24 h after surgery with rest. Among these studies, one study divided the gabapentin group into two groups according to whether the gabapentin was administered preoperatively or postoperatively; thus, a total of six clinical studies were included. Our meta-analysis revealed that gabapentin produced no better outcome than placebo in terms of VAS scores with rest at 24 h (SMD = 0.15; 95 % CI −0.17 to −0.48; *P* = 0.360, Table 2) and with rest at 48 h (SMD = 0.22; 95 % CI −0.25 to 0.69; *P* = 0.363, Table 2).

#### VAS score with mobilisation

A total of five studies (451 patients) provided VAS scores at 24 h with postoperative mobilisation. There was no statistically significant difference between the groups with respect to the VAS scores at 24 h postoperatively (SMD = 0.46; 95 % CI −0.19 to 1.11; *P* = 0.164, Table 2). Only three studies with 331 THAs reported the VAS score at 48 h postoperatively; our meta-analysis found no significant difference between the two groups (SMD = 1.15; 95 % CI −0.58 to 2.89; *P* = 0.193, Table 2).

#### Complications

**Fig. 5:**
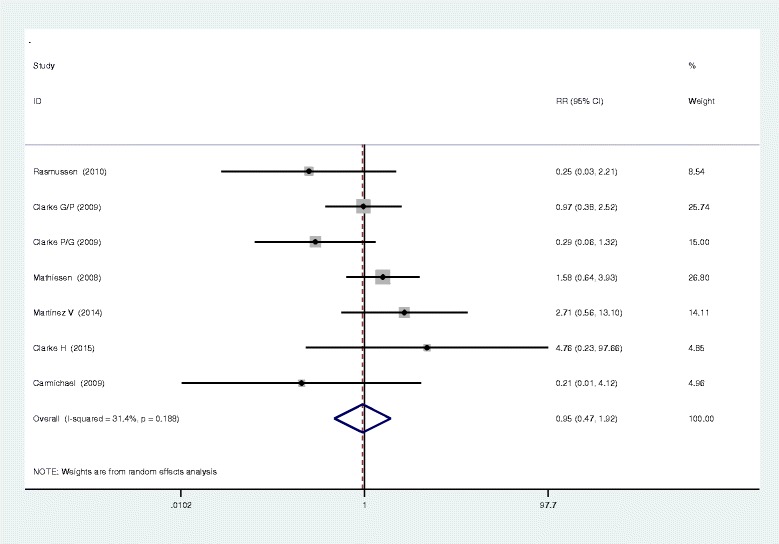
The forest plot of occurrence of vomiting between the two groups

**Fig. 6:**
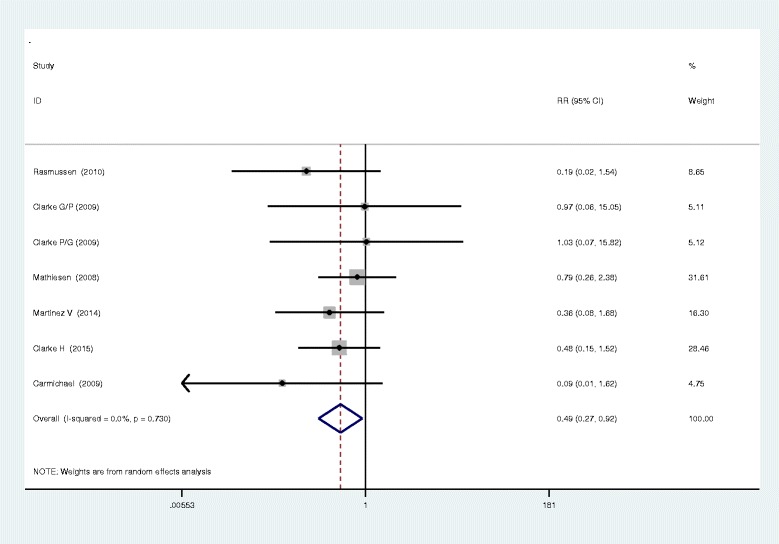
The forest plot of occurrence of nausea between the two groups

**Fig. 7:**
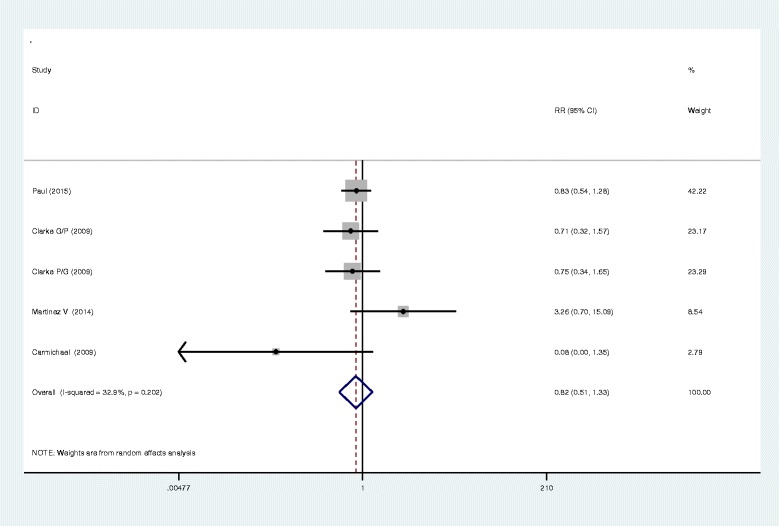
The forest plot of occurrence of dizziness between the two groups

**Fig. 8:**
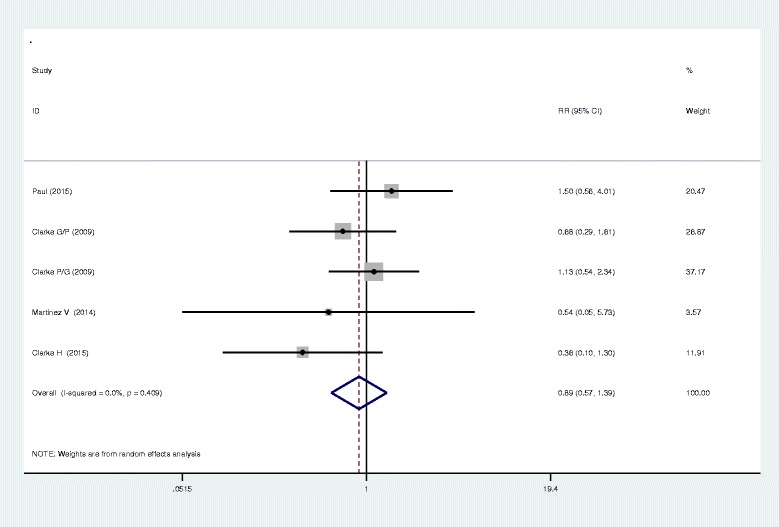
The forest plot of occurrence of pruritus between the two groups

Seven studies closely monitored postoperative vomiting. Our meta-analysis identified no significant difference between the two methods in terms of postoperative vomiting (RR, 0.95; 95 % CI 0.47–1.92, *P* = 0.895, Fig. 5), with a low heterogeneity (*I*^2^ = 31.4 %, *χ*^2^ = 7.30). Six studies investigated the occurrence of nausea in both methods and found that the administration of gabapentin or pregabalin can increase the occurrence of nausea (RR, 0.49; 95 % CI 0.27–0.92, *P* = 0.025, Fig. 6). In addition to the above complications, there was no statistically significant difference between the incidence of dizziness and pruritus (RR, 0.82; 95 % CI 0.51–1.33, *P* = 0.429; RR, 0.89; 95%CI 0.57–1.39, *P* = 0.600, Figs. 7 and 8).

## Discussion

To our knowledge, this is the first meta-analysis of RCTs comparing the efficacy and safety of gabapentin or pregabalin with placebo for the management of pain after THA. The present meta-analysis was conducted on the basis of seven randomised studies that found lower cumulative morphine consumption at 24 h and 48 h postoperatively with gabapentin or pregabalin administration than with placebo. There was no significant difference between the two groups with mobilisation at 24 h or 48 h. In addition, perioperative gabapentin administration can decrease the occurrence of nausea; however, there is no significant difference between the two groups in terms of incidence of vomiting, dizziness and pruritus.

The results of our meta-analysis indicated that preoperative gabapentin or pregabalin can decrease the cumulative morphine consumption at 24 h and 48 h, and the difference is statistically significant. The management of acute pain after THA has been well studied and involves multimodal analgesia, including opioids and other adjuncts, such as dexamethasone, morphine, pregabalin and gabapentin [12, 16, 18, 19]. The two main purposes of balanced analgesia are to improve analgesia and reduce the complications. A large number of trials have suggested limited or no benefits of pregabalin or gabapentin for acute pain and a significant reduction in pain control after surgery [20–22]. Gabapentin was first introduced as an antiepileptic drug in 1993 and has since been used to treat painful neuropathies. Pregabalin was introduced as an anticonvulsant in 2004 [23]. Compared with gabapentin, pregabalin possesses superior oral absorption and bioavailability; thus, pregabalin has attracted much interest as an adjunct in the management of neuropathic and postoperative pain.

There is no significant difference between the VAS scores at 24 and 48 h with rest or mobilisation. However, there is not enough data to compile for VAS scores from operation to 24 h. Subgroup analysis indicated that neither gabapentin nor pregabalin is superior to placebo. The primary mechanism of gabapentin action is achieved in combination with the 21 subunits of presynaptic voltage-gated calcium channels. The expression of these channels is upregulated upon nerve injury. Furthermore, gabapentin can decrease the hyperexcitability of secondary nociceptive neurons in the dorsal horn. The relevant VAS score in the included studies were reported only for 24 and 48 h, and the long-term pain control effect is unknown. Hence, studies on the long-term effect of gabapentin or pregabalin for pain control after THA are needed. Yao et al. [24] conducted a meta-analysis to compare preoperative pregabalin for pain control in gynaecological surgery and found that it has analgesic and opioid-sparing effects and does not increase the frequency of adverse effects. Eipe et al. [25] conducted a meta-analysis to compare the effects of pregabalin for acute pain and concluded that the analgesic effectiveness is largely restricted to surgical procedures. Another reason may be that all the studies involved administration of postoperative analgesia in the form of patient-controlled analgesia. This method achieves better pain control; thus, additional gabapentin or pregabalin are not needed to achieve better pain control after THA.

Gabapentin or pregabalin can decrease the occurrence of nausea without increasing the complications of vomiting, pruritus and dizziness with a low heterogeneity. Morphine-related complications, including pruritus and dizziness, especially dizziness, may prolong the length of hospital stay and thus increase the economic burden. Since gabapentin or pregabalin can decrease the cumulative morphine consumption, this is one of the reasons for the decrease in the incidence of nausea. Another point of clinical significance is that gabapentin can successfully reduce opioid consumption and thus can lead to more stable haemodynamics and reduced respiratory depression. Since the sample size is limited, the other complications did not reach statistical significance. Therefore, more high-level RCTs are needed to further identify the complications of gabapentin or pregabalin for THA. In this meta-analysis, patient-reported outcomes were not specifically assessed. Two studies reported the Western Ontario and McMaster university Osteoarthritis Index (WOMAC) score; however, this score did not show a significant difference between pregabalin or gabapentin versus placebo in the two studies [11, 12].

There were several limitations in this meta-analysis: (1) only seven RCTs were included, and sample sizes of the included studies were relatively small, which might have affected the precision of the effect size estimations.; (2) we only included studies with immediate follow-up at 24 and 48 h postoperatively; (3) the dose and time of gabapentin or pregabalin differed between the studies, which will affect the precision of the results; (4) the multiple analgesia approaches are different from each other, and consistent multiple analgesia approaches are needed to identify the most effective pain control method; and (5) even though the Begg’s test provides evidence of funnel plot symmetry indicating that there is no publication bias, we cannot completely exclude publication bias because the number of the studies included was limited.

## Conclusions

In conclusion, although the number of studies and samples in each paper is limited, this is the first meta-analysis that compares the use of gabapentin or pregabalin with a placebo for the management of pain after THA. Our meta-analysis revealed that gabapentin has an analgesic- and opioid-sparing effect in acute postoperative pain management without increasing the incidence of nausea. Because the sample size and number of included studies is limited, a multiple central randomised controlled trial is needed to identify the effects and optimal dose of gabapentin for reducing pain after THA.

## Additional file

Additional file 1: Search strategies. (DOCX 145 kb)

## Abbreviations

CIs: Confidential intervals
DVT: Deep venous thrombosis
MD: Mean difference
NSAIDs: Nonsteroidal anti-inflammatory drugs
RR: Relative risk
THA: Total hip arthroplasty
VAS: Visual analogue scale
WOMAC: Western Ontario and McMaster University Osteoarthritis Index
