# Supplementary material for: The efficacy of preoperative administration of gabapentin/pregabalin in improving pain after total hip arthroplasty: a meta-analysis
Source: BMC Musculoskelet Disord. 2016 Aug 30;17(1):373. doi: 10.1186/s12891-016-1231-4 (PMC5004259; doi:10.1186/s12891-016-1231-4)
Supplement: Additional file 1: — Search strategies. (DOCX 145 kb) [file 12891_2016_1231_MOESM1_ESM.docx]

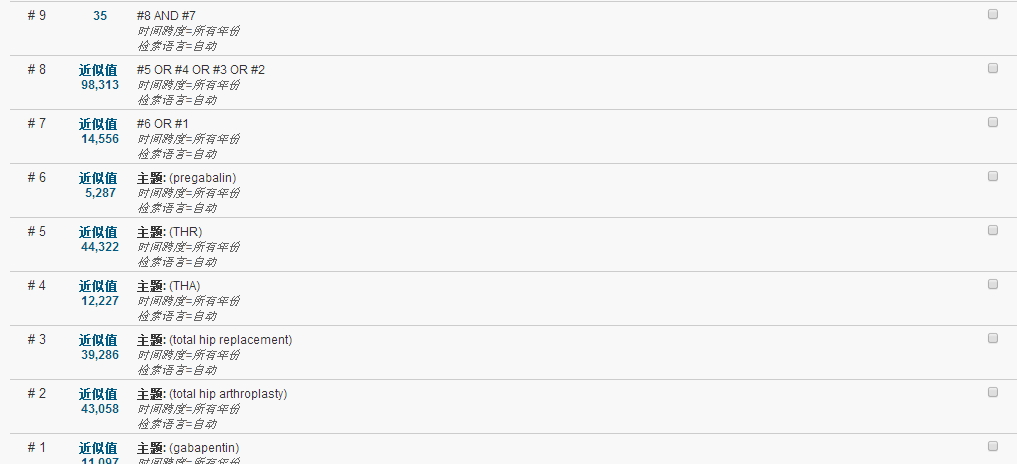


Database: Web of Science

#1 gabapentin

#2 total hip arthroplasty

# 3 THA

#4 total hip replacement

#5 THR

#6 pregabalin

#7 #6 OR #1

#8 #5 OR #4 OR#3 OR #2

#9 #8 AND #7
